# History of global burden of disease assessment at the World Health Organization

**DOI:** 10.1186/s13690-020-00458-3

**Published:** 2020-08-24

**Authors:** Colin D. Mathers

**Affiliations:** 1 Consultant on Global Health, Geneva, Switzerland; 2 College of Medicine & Veterinary Medicine, University of Edinburgh, 30 West Richmond Street, Edinburgh, EH8 9DX UK

**Keywords:** Global burden of disease, World Health Organization, Global health statistics, IHME, WHO, Sustainable development goals

## Abstract

**Background:**

The World Health Organization collaborated in the first Global Burden of Disease Study (GBD), published in the 1993 World Development Report. This paper summarizes the substantial methodological improvements and expanding scope of GBD work carried out by WHO over the next 25 years.

**Methods:**

This review is based on a review of WHO and UN interagency work relating to Global Burden of Disease over the last 20 years, supplemented by a literature review of published papers and commentaries on global burden of disease activities and the production of global health statistics.

**Results:**

WHO development of global burden of disease work in the Millenium Development Goal era resulted in regular publication of time series estimates of deaths by cause, age and sex at country level, consistent with UN population and life table estimates, and with cause-specific statistics produced across UN agencies and interagency collaborations. This positioned WHO as the lead agency to monitor many of the 43 health-related indicators for the UN Sustainable Development Goals.

In 2007, the Institute of Health Metrics and Evaluation (IHME) was established to conduct new global burden of disease and related work, funded by the Bill and Melinda Gates Foundation (BMGF). WHO was a core collaborator in its first GBD2010 study, but withdrew prior to publication as it was unable to obtain full access input data and methods. The publication of global health statistics by IHME resulted in user confusion and in debate over differences and the reasons for them. The new WHO administration of Director General Dr. Tedros Ghebreyesus has sought to make greater use of IHME outputs for its global health statistics and SDG monitoring.

**Conclusions:**

WHO work on global burden of disease has positioned it to be the lead agency for monitoring many of the UN Sustainable Development Goals. Current moves to use IHME analyses raises a number of issues for WHO and for Member States in relation to WHO’s constitutional mandate, its accountability to Member States, the consistency of WHO and UN demographic and health statistics, and the ability of Member States to engage with the results of the complex and computer-intensive modelling procedures used by IHME. As new global health actors and funders have arisen in recent decades, and funding to carry out WHO’s expanding mandate has declined, it is unclear whether WHO has the ability or desire to continue as the lead agency for global health statistics.

## Background

The production and dissemination of health information for priority setting and assessment of progress at the country, regional and global levels are core activities of the World Health Organization (WHO) mandated by the Member States in the Constitution. Starting with the first Global Burden of Disease (GBD) study in the early 1990s, WHO has seen a consistent and comparative description of the burden of diseases and injuries, and the risk factors that cause them, as an important input to health decision-making and planning processes.

From the early 2000s, the emphasis on country-level tracking of progress towards agreed global health targets has increased WHO’s investments in statistical monitoring across all its priority areas, and has spurred the creation of interagency collaborations to harmonize health statistical work across relevant UN agencies. At the same time, the increasing numbers of actors in the global health field, and particularly the Bill and Melinda Gates Foundation with its funding of an academic group conduct Global Burden of Disease work, has led to a situation where the global health community is faced with multiple inconsistent sets of global health statistics. This paper reviews the history of global burden of disease assessment at WHO and issues for its future role in global health statistics.

## Methods

From 2002 to 2018, I had management responsibility for WHO work on global burden of disease as well as responsibility for the overall quality and clearance of WHO official health statistics. This review of the work of WHO on global burden of disease is based on a literature review of WHO publications on burden of disease, and published papers and commentaries on global burden of disease activities and the production of global health statistics. It is also supplemented by my extensive knowledge and involvement in WHO and UN interagency work in this area over the last 20 years.

## Results

### The first global burden of disease study in the 1990s

The original GBD study was commissioned in 1992 by the World Bank for its 1993 World Development Report on *Investing in Health* recommended cost-effective intervention packages for countries at different levels of development [1]. Underpinning these analyses was the first Global Burden of Disease (GBD) study, carried out by Chris Murray at Harvard University and Alan Lopez at the World Health Organization (WHO), in collaboration with a global network of over 100 scientists [1–3]. This first GBD study quantified the health effects of more than 100 diseases and injuries for eight regions of the world in 1990. Earlier attempts to quantify global cause of death patterns [4] had been largely restricted to broad cause of death groups.

The study also introduced a new metric – the disability-adjusted life year (DALY) – as a single measure to quantify the burden of diseases, injuries and risk factors (Murray, 1996). The DALY is a composite measure that adds YLL, years of life lost from premature death, and YLD, years of “equivalent healthy life” lost (YLD) through living in states of less than full health [2]. The DALY naturally weights deaths at younger ages more heavily, but also explicitly included time discounting (of future years of life lost) and age-weighting (lower weight for younger and older years of life).

The results of the original GBD study were surprising to many health policy makers, more familiar with the pattern of causes represented in mortality statistics. For example, the leading causes of disease burden in 1990 included childhood diseases, mental disorders and road traffic accidents. The study was also controversial for a number of reasons: the substantial use of imputation and modelling due to lack of data in some regions and for many causes [5], the use of disability weights to equate years lived with disability or functioning limitations to loss of years of full health [6], and the use of discounting and age weights [7]. Although the first two volumes of the GBD published in 1996 [2, 3] cited eight additional books providing information on cause-specific data and methods, only two further volumes were ever published in 1998 and 2004 [8, 9].

### GBD 2000–2004 at the World Health Organization: improved methods, more data

When Dr. Gro Harlem Brundtland took office as Director-General of WHO in July 1998, she also brought Chris Murray to WHO to provide a stronger focus on evidence for health policy. An update of the GBD for the year 1999 was a major input to the assessment of healthy life expectancy for WHO Member States, used as one of the outcome measures to quantify health system performance and rank countries in the *World Health Report 2000* [10]. From 1999 to 2004, the World Health Organization (WHO) published an annual update of the GBD in the *World Health Report* Annex tables. An expanded research program was undertaken to quantify the global and regional attributable mortality and burden for 26 major risk factors. It was released in the *World Health Report 2002* [11] and in subsequent detailed volumes in 2004 [12].

The GBD results for the year 2001 provided a framework for cost effectiveness and priority setting analyses carried out for the Disease Control Priorities Project (DCPP), a joint project of the World Bank, WHO and the National Institutes of Health [13]. The GBD results were documented in detail, with information on data sources and methods as well as uncertainty and sensitivity analyses, in a book published as part of the DCPP [14].

During this period, new tools were developed and used for the GDB updates: a new system of model life tables, new standardized methods to predict adult mortality from child mortality where information on adult mortality was not available, and a new software tool, DISMOD II, for ensuring epidemiological consistency of incidence, prevalence and disease-specific mortality for DALY calculations [15].

### GBD at the World Health Organization 2005–2009

Following the departure of Chris Murray from WHO after the election of Dr. J. W. Lee as Director General of WHO in 2003, the author continued to lead work on the updating of the GBD at WHO. This led to the publication of updated projections of cause-specific mortality and burden of disease from 2002 to 2030 [16] and to comprehensive updates of the GBD for the year 2004 [17] and of comparative risk assessment for 24 global health risks [18]. These were followed by an updated assessment of deaths by cause for the year 2008 [19].

By the time of the GBD 2004 study, mortality and cause of death estimates had been completely updated using new tools and data from the WHO Mortality Database and from censuses, surveys, epidemiological studies and surveillance systems [17]. For the estimation of YLD, years lost due to disability from diseases and injuries, 97 of the 136 disease and injury causes had been updated, including all causes of public health importance or with significant YLD contribution to DALYs. Disability weights were the main area where a comprehensive update was not carried out, though quite a few were revised using a European study [20] and other sources of population information on health states. To address criticisms about lack of transparency in the original GBD analyses, substantial effort was also put into documenting cause-specific analyses and the overall analytical approach [12, 14, 17, 18].

The adoption of the Millenium Development Goals (MDGs) by the United Nations General Assembly in 2000 and the development of targets for the year 2015 during the early years of the twenty-first century, including 12 health targets, resulted in substantial effort by WHO and other UN Agencies to measure and monitor trends for these targets, including key indicators for child and maternal mortality, HIV, malaria and tuberculosis. In the early 2000s, several UN agencies were independently estimating some of these key indicators. For example, when WHO started making its own estimates of child mortality under Chris Murray, there were four different sets of country-level child mortality rates published by international agencies (UN Population Division, UNICEF, World Bank and WHO).

The Interagency Group on Child Mortality Estimation (UN-IGME) was established in 2004, to produce a common set of child mortality estimates for the UN agencies [21]. This was followed by the Maternal Mortality Estimation Inter-Agency Group [22] and other WHO interagency collaborations for various diseases, injuries and risk factors. The GBD2004 thus drew quite heavily on UN interagency estimates in a number of areas and also provided a comprehensive context for the MDG health targets and indicators.

In part because the GBD analyses at WHO had moved to countries rather than regions as the unit of analysis, and because of the country-level focus of MDG reporting, WHO started reporting GBD results at country rather than regional level for the GBD 2004. This also motivated increased engagement with Member States not only through formal consultation but also through increased emphasis on improvement of national health information systems through initiatives such as the Health Metrics Network [23].

Responding to the increased interest among researchers and national health authorities in carrying out national burden of disease (NBD) studies, WHO produced a software package which allowed a national study team to generate a system of interlocking spreadsheets filled with prior estimates of deaths, incidence, prevalence, YLD and DALYs for their country drawn from the WHO GBD national-level results. The NBD team could then revise these estimates for any diseases, injuries and disabilities for which national data were available and immediately generate a comprehensive updated national study [24].

After Murray left WHO in 2003 and returned to Harvard, he argued for an independent agency outside WHO to take on the role of producing unbiased and objective information on population health [25]. Despite his previous efforts to institutionalize the production of such global health statistics within WHO, he and coauthors claimed that WHO has an inherent conflict of interest in relation to the production of unbiased and objective information on population health. They argued that the very WHO departments undertaking such syntheses are also the departments whose performance is being judged on the basis of such information, and that WHO is under pressure from Member States to accept and use biased and inaccurate data.

WHO pointed to its constitutional mandate to provide Member States with sound unbiased technical advice, its accountability to Member States, its ability to mobilize global expertise, and its unique position to generate productive engagement between global monitoring and country information systems [26, 27]. It also emphasized the commitment of WHO to transparent processes, reproducible methods, and country consultation and involvement. WHO also seeks improved collaboration with academic research groups and for productive and healthy debates on improving data and methods for global health statistics.

Public release of country-level estimates by WHO is preceded by consultation with its Member States [28]. The consultation gives Member States an opportunity to comment on methods and data sources and to provide updated input data and typically lasts 1 month. Since WHO aims to use a standard and comparable methodology for all countries, and that known measurement biases be adjusted in the estimation process, it is often the case that WHO and interagency estimates are not identical with official national estimates. For WHO official statistics released at Member State level, there is a disclaimer indicating that WHO statistics may differ from official national statistics due to differences in data and methods.

The country consultation process offers a valuable opportunity to maximize consistency of data inputs and assumptions, and to improve understanding of reasons for differences. This process has been further facilitated by increasing use of multi-country workshops and regional consultation meetings, together with increased coordination and involvement of WHO regional and country offices.

During the period 2003 to mid-2017, under three WHO Director Generals, I was responsible for statistical clearance of all health statistics published by WHO. During that period, there were certainly occasions on which technical Departments interpreted scientific data in ways that aimed to emphasise achievement (lower numbers) or facilitate advocacy for action and funding (higher numbers) and revisions were required before statistics were released. There was also substantial pressure on occasion from some Member States, large and small, to alter statistics and there was only one instance in which senior management did not fully back the use of standardized data and methods by WHO, and resist country pressure. That one occasion related to a specific statistic for a country for which WHO had no data, and had made use of an imputed value from IHME, which it was not able to fully defend.

But it is quite true that the independence of WHO technical advice depends crucially on the commitment of senior staff and the Director General and their willingness to resist political pressure. In exactly the same way, the independence of IHME is crucially dependent on the willingness of its Director to resist pressure from BMFG and other important stakeholders.

### WHO collaboration with IHME for the GBD2010

In 2007, the Institute of Health Metrics and Evaluation (IHME) was established in Seattle under the leadership of Chris Murray to carry out new global burden of disease work and to assess the performance of health programs around the world. IHME was funded by a 10 year grant of $105 M from the Bill and Melinda Gates Foundation (BMGF). The GBD2010 study was set up as a collaboration between IHME and Harvard University, the World Health Organization, Johns Hopkins University and The University of Queensland, as well as drawing on the expertise of around 40 expert working groups [29].

The GBD2010 Core Team was established in 2007 as the central scientific decision-making body for the GBD2010, with membership from the GBD collaborating institutions. Nine of the fifteen original members were from outside IHME, including Ties Boerma and the author from WHO. Over the next 5 years, the author and many other WHO staff contributed to the work of the GBD, though with growing concern that external Core Team members and collaborating WHO staff were being excluded from access to the data and analyses.

Around the period 2011 to 2012, five of the external Core Team members withdrew from the Core Team as IHME refused to allow access to primary data, data sources or involvement in crucial methodological decisions.. From WHO’s point of view, there was no cold war with IHME as later claimed by Richard Horton, editor of the Lancet [30]. The Lancet has been a key platform for the publication and dissemination of GBD results. The annual paper series on the latest GBD update get priority treatment, with reviewers often being requested to give rapid review within days or a week.

Various WHO staff continued to provide data and contribute to GBD analyses, and WHO continued to make use of analyses derived from the IHME GBD results. However, because WHO could not gain access to GBD2010 input data and analyses, WHO staff were unable to agree to be authors on GBD papers and WHO as an institution did not endorse the results. Perhaps more importantly, WHO was also unable to properly examine areas where GBD results differed from WHO and other UN statistics in order to reconcile differences and potentially improve global health statistics. The WHO Director General still welcomed the GBD2010 study as an unprecedented effort to improve global and regional estimates of levels and trends in the burden of disease [31].

While many of the GBD2010 results were similar to recent estimates of WHO, there were sometimes large differences. For example, the GBD2010 study estimated that there were 1.24 million deaths due to malaria in 2010, with more than half a million of these occurring in those aged 5 years and older [32]. These estimates were substantially larger than those of WHO at the time: 655,000 deaths in total, with less than 100,000 in those aged 5 years and over [33]. There was strong criticism of the IHME estimates from some malaria experts [34] and a WHO Expert Meeting identified IHME methods for interpretation of verbal autopsy data as the reason for their high estimates of adult malaria deaths. IHME also estimated less than 200,000 child tuberculosis cases in 2013, much less than the approximately 350,000 cases notified to WHO in 2012, and substantially less than the WHO estimate of 530,000 cases [35]. IHME estimates of all-cause mortality rates and time trends varied substantially in some cases from those of the United Nations Population Division (UNPD). For example, the GBD2010 estimated there were 817,000 deaths for children aged 5–14 years in 2010, only 57% of the 1.44 million estimated by UNPD [36]. Later analyses of survey data resulted in estimates of global deaths for children aged 5–14 year that were much closer to the UNPD figure [37].

With its focus on vaccine-preventable diseases in children, the BMGF was particularly concerned about differences in IHME and WHO estimates for global deaths from these diseases. It convened a meeting of IHME, WHO, CHERG (WHO expert advisory group) and independent experts to assess reasons for differences. The largest difference was for pneumonia deaths in children aged under 5 years where CHERG/WHO estimated 1.4 million deaths in 2010, compared to 0.85 million by IHME. Differences were identified as due to differences in estimates of total under 5 deaths, GBD2010 broader inclusion criteria for verbal autopsy and death registration data, differences in cause attribution hierarchies used, and in the use by IHME but not CHERG of observational studies to attribute pneumonia aetiology as well as vaccine efficacy studies used by both groups [38].

A meeting convened by BMGF recommended that the impact of different data inputs be examined by applying the analytic methods of both groups and systematically excluding classes of data in their models. For its next GBD update released in 2014 before the publication of the expert review of differences [38], IHME changed its method of attributing deaths to pathogens to a counterfactual approach. The comparative analyses of differences due to methods and to data included were not carried out as IHME continued to be unwilling to provide access to its input datasets or modelling software.

### WHO Global Health estimates 2013 to present

Starting in 2013, WHO released regularly updated time series estimates of deaths and DALYs by cause, age and sex for WHO Member States [39, 40]. These were rebranded as WHO Global Health Estimates (GHE) to avoid confusion with the IHME GBD estimates. The GHE addressed the increasing demand for time-series, including monitoring of progress towards MDGs and other health targets, for country-level results, and for comprehensive estimates across non-communicable disease and injury causes. With the move to GHE, WHO ceased to use the model life table system developed in the early 2000s, and the WHO life table time series for countries were brought into alignment with the UN Population Division biennial World Population Prospects to the extent possible, with adjustments to maximize consistency with UNAIDS estimates for HIV mortality and with national death registration data collected in the WHO Mortality Database [41].

Table 1 lists the various GBD and GHE versions carried out by WHO as well as the original GBD 1990 study, which was a collaboration between Harvard University, WHO and the World Bank.

**Table 1:** Summary of global burden of disease estimates prepared by WHO or in collaboration with WHO from 1993 to 2020

Version	Year of publication	Years of estimates	Geographic level of		# of age			
			Analysis	Publication	groups	Risk factors	Projections	Reference
GBD 1990	1993, 1996	1990	6 regions	6 regions	5	10	2000, 2010, 2020	[2, 3]
GBD 2000	2002	2000	14 subregions	14 subregions	8	26		[10–12]
GBD 2001	2006	2001	192 MS*	Regional groupings	8	26	2002–2030	[14, 16]
GBD 2002	2004	2002	192 MS	Country-level	8			[42]
GBD 2004	2008, 2009	2004	192 MS	Country-level		24	2004–2030	[17, 18]
COD 2008	2011	2008	193 MS	Country-level				[19]
GHE 2011	2013	2000–2011	193 MS	Regional groupings				[39]
GHE 2012	2014	2000–2012	194 MS	Country-level	20	selected		[39, 40]
GHE 2015	2016	2000–2015	194 MS	Country-level	20	selected	2015–2030	[39, 40]
GHE 2016	2018	2000–2016	194 MS	Country-level	20	selected	2016–2060	[39, 40]
GHE 2019	In progress	2000–2019	194 MS	Country-level	20			

These WHO GHE provide a comprehensive and comparable set of cause of death estimates from year 2000 onwards, consistent with and incorporating UN agency, interagency and WHO estimates for population, births, all-cause deaths and specific causes of death, including HIV, tuberculosis, malaria, maternal mortality, major causes of child death, cancers, road traffic accidents, homicides and conflicts and natural disasters. Ensuring consistency across cause analyses that are created by various agencies and groups is more difficult than for comprehensive estimates that are prepared by a single academic group. This is offset by the advantage of having a comprehensive context for disease, injury and risk factor specific analyses and advocacy that is largely consistent with estimates for those topics published in WHO flagship reports and by other UN agencies, as an input to the assessment of achievement of the MDGs in 2015, and for assessment of the likely issues in addressing and achieving SDG health targets [43].

**Fig. 1:**
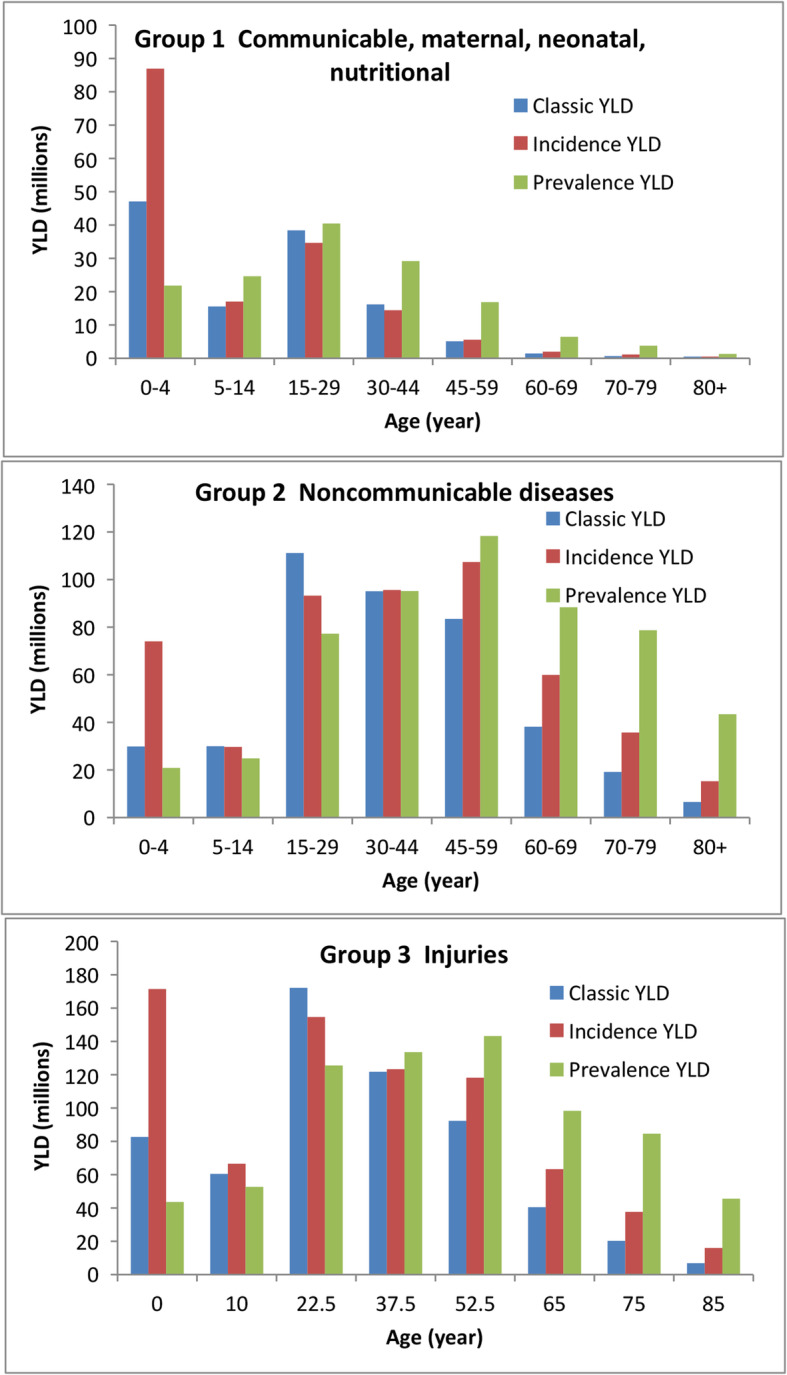
Effect of change in definition of YLD on the age distribution of global YLD for the year 2004. Classic YLD are incidence-based with age-weighting and 3% time discounting; incidence and prevalence YLD are not age-weighted or discounted. Source: [44]

To meet the need for DALY estimates consistent with the GHE estimates of cause-specific mortality, WHO has also released regular updates for DALY time series by cause, age and sex for years 2000 onwards. These also draw on IHME GBD analyses for YLDs for most causes, with some revisions and methodological differences as summarized below [44]:

- A simpler form of DALY has been adopted in line with decisions taken for the GBD2010 study. Age-weighting and time discounting are dropped, and the YLDs are calculated from prevalence estimates rather than incidence estimates (see Fig. 1). YLDs are also adjusted for independent comorbidity.
- The standard life table used for calculation of years of life lost for a death at a given age is based on the projected frontier life expectancy for 2050, with a life expectancy at birth of 92 years. The same frontier life table is used for males and females, unlike earlier versions of the GBD.
- The years of life lost from mortality (YLLs) are calculated using WHO estimates of deaths by cause, age and sex.
- Estimates of YLD draw on the IHME GBD analyses, with selected revisions to disability weights and prevalence estimates as noted below.
- Limited revisions have been made to disability weights for infertility, intellectual disability, vision loss, hearing loss, dementia, drug use disorders and low back pain.
- WHO estimates of vision and hearing loss prevalence by country and their cause distributions have been used to calculate YLDs for vision and hearing loss sequelae. Revised severity distributions have been taken into account in estimating YLDs for migraine, back/neck pain and skin disorders.
- The GBD did not include problem use as a sequela for alcohol use disorders as was done in the GBD 2004. YLDs for problem use of alcohol have been estimated and added to the YLDs for alcohol dependence.

With the increased focus on MDG and SDG health targets over the last two decades, WHO and other UN agencies have become much more focused on cause-specific and total mortality rates, as well as various incidence and prevalence indicators, rather than YLD or DALYs. The DALY summary measure has not been seen as greatly relevant, even via computation of healthy life expectancy summary measures, which both WHO and IHME unsuccessfully promoted as a desirable overarching indicator of health progress for the SDGs [45]. Thus, the WHO GHE program has focussed primarily on estimates of cause-specific deaths, and largely drawn estimates of YLD from IHME analyses.

The WHO GHE program has played an important role in positioning WHO to monitor the 43 health-related indicators in the UN Sustainable Development Goals (see Table 2). WHO is currently the lead agency for 27 of these indicators, and a partner in interagency groups for another seven [46]. The WHO Global Health Statistics are currently the source for WHO reporting on eight of the SDG health indicators, and of course, a number of others feed into GHE updates. The 2018 World Health Statistics [47] reported country-level values for 36 of the health-related SDG indicators along with analyses of trends. The GHE estimates have also provide a context for placing the SDG target areas in a comprehensive context for all diseases and injuries, disaggregated by age, sex, country and year.

**Table 2:** WHO and GHE role in monitoring and reporting on SDG health-related targets

Indicator	Indicator area	Lead agency	Source of statistics published in WHO World Health Statistics
3.1.1	Maternal mortality	WHO	MMEIG Interagency Group
2.1.2	Skilled attendants at birth	UNICEF	WHO-UNICEF Interagency Group
3.2.1	Under-five mortality rate	UNICEF	UN-IGME Interagency Group
3.2.2	Neonatal mortality rate	UNICEF	UN-IGME Interagency Group
3.3.1	HIV incidence	UNAIDS	UNAIDS/WHO
3.3.2	Tubeculosis incidence	WHO	WHO technical program
3.3.3	Malaria incidence	WHO	WHO technical program
3.3.4	Hepatitis B incidence	WHO	WHO technical program
3.3.5	Need for neglected tropical disease interventions	WHO	WHO technical program
3.4.1	NCD mortality	WHO	WHO GHE
3.4.2	Suicide	WHO	WHO GHE
3.5.1	Treatment for substance use disorders	UNODC	
3.5.2	Alcohol use	WHO	WHO technical program
3.6.1	Road traffic mortality	WHO	WHO technical program & GHE
3.7.1	Family planning	UN Pop. Division	UN Pop. Division
3.7.2	Adolescent birth rate	UN Pop. Division	UN Pop. Division
3.8.1	UHC service coverage index	WHO	WHO technical program
3.8.2	UHC financial protection	WHO	WHO technical program
3.9.1	Mortality due to air pollution	WHO	WHO technical program & GHE
3.9.2	Mortality due to unsafe WASH services	WHO	WHO technical program & GHE
3.9.3	Mortality due to unintentional poisoning	WHO	WHO GHE
3.a.1	Tobacco use	WHO and FCTC	WHO technical program
3.b.1	Vaccine coverage	WHO	WHO technical program
3.b.2	Development aid for health	WHO	WHO technical program
3.b.3	Essential medicines	WHO	
3.c.1	Health workforce	WHO	WHO technical program
3.d.1	IHR capacity and emergency preparedness	WHO	WHO technical program
1.a.2	Gov’t spending on services	–	WHO technical program
2.2.1	Child stunting	UNICEF	WHO-UNICEF-World Bank
2.2.2	Child malnutrition	UNICEF	WHO-UNICEF-World Bank
5.2.1	Intimate partner violence	WHO	
5.2.2	Sexual violence by others (non int. partner)		
5.3.2	Female genital mutiliation	UNICEF	
6.1.1	Safe water	WHO	WHO-UNICEF Joint Monitoring Program
6.2.1	Safe sanitation	WHO	
6.3.1	Treated wastewater	WHO	
7.1.2	Reliance on clean fules	WHO	WHO technical program
11.6.2	Fine particulate matter	WHO	WHO technical program
13.1.2	Deaths due to disasters	–	WHO GHE
16.1.1	Homicide	WHO	WHO GHE
16.1.2	Conflict deaths	–	WHO GHE
16.1.3	Population subjected to violence	–	
17.19.2	Birth and death registration	WHO	WHO and UNDESA

### WHO collaboration with IHME after 2012

During the period following the release of the Global Burden of Disease 2010 study at the end of 2012 by IHME, WHO continued to seek active collaboration with IHME in the production of global health statistics. In 2013, WHO established an overarching Reference Group on Health Statistics (RGHS) to provide advice and guidance on priorities for WHO work in health statistics and invited Chris Murray and Rafael Lozano of IHME join the group [48]. Other staff of IHME also participated in later meetings.

The publication of global statistics by IHME created a new situation in which users had a “choice” of estimates − from the UN system and from academia. For many areas of difference, WHO and others sought more detailed information on IHME methods and access to the input data used in order to evaluate these differences and where appropriate revise and improve their statistics and methods, as well as to be able to advice Member States and the public on the reasons for differences, and caveats for use. A common experience was to find that IHME input data was not obtainable, and in some cases, that only broad descriptions of methods were available. For example, Alkema et al. [49] carried out a comparison of UN-IGME and IHME estimates for child mortality and commented that “we were unable to further examine the exact causes of discrepancies for each country between the two sets of estimates since the IHME database is not publicly available. Responding to repeated requests from the authors to share the data, IHME researchers and staff pointed out that researchers were occupied producing the Global Burden of Disease estimates and would not have time to make the data available until the academic papers on this topic were submitted. No projected date has been given so far for sharing the data …. , ten months after the IHME’s paper on child mortality was published.”

WHO had some similar experiences in seeking sufficient information to evaluate differences in estimates, but in other cases, dialogue with IHME resulted in clarification of differences and in some cases improvements in data and statistical methods used by both IHME and WHO. For example, the ensemble regression modelling concept introduced by IHME for the GBD2010 was adopted by WHO for making estimates of homicide rates [50] and in other areas.

There are a large number of significant differences between the GBD and WHO/UN statistics. Reasons for some of these are known, others probably arise from differences in input data and its adjustments, in the methods used for interpreting verbal autopsy and sibling survival data reported in surveys, and in the complex modelling procedures used. Examples include differences in maternal mortality trends in Africa arising from differences in total reproductive age mortality associated with interpretation of sibling survival data in surveys [51], and an IHME overestimate of road injury mortality in Europe arising from an inappropriate redistribution of deaths assigned cause “unknown accident” [52]. Some differences persist across revisions of GBD, others arise and disappear across revisions as methods and data change. Increasing concerns about the transparency and replicability of global health estimates, and difficulties in assessing reasons for differences, led the RGHS to set up a working group in 2014 to define and promote best practice in reporting health estimates, with active involvement from WHO, IHME and others involved in publication of health research or the development of publication guidelines. This resulted in a consensus statement reporting list known as GATHER, published in 2016 simultaneously in the Lancet and PLoS Medicine [53], together with commitments from WHO and IHME to implement GATHER standards. In 2015, WHO and IHME signed a Memorandum of Understanding (MoU) to increase cooperation and to facilitate data and information exchange [54].

IHME has taken steps to implement the GATHER guidelines, with more detailed cataloguing and publication of data in the GHDx database [55], release of computer code and increased levels of documentation. This has improved transparency considerably, but it remains the case that full replication even of specific results is in practice not possible. Apart from the requirement for computing resources and staffing beyond the reach of most academics or health authorities, IHME input datasets for analyses are not usually in the public domain, or even available to collaborators. IHME has argued that this is because some data is confidential, but it has refused to make available to UN and WHO analytic groups relevant datasets excluding the confidential data. It would be an important first step in assessing reasons for differences in estimates to determine whether non-public data makes substantive differences to results. Addiionally, input data sets have been through processes to adjust for known biases, to impute missing data, to cross-walk to standard definitions, and also weighted in various ways relating to data quality. These processes are not usually fully documented or replicable.

WHO has continued to provide data to IHME, to collaborate in a number of areas, and to assess and make use of IHME GBD results to the extent possible. Over time, some convergence has occurred between GBD and WHO estimates, although major differences remain in areas such as adult malaria mortality. However, until recently WHO has been unwilling to rely completely on statistics for which it is not responsible or accountable to member states and for which it does not have, in many cases, full access to the data and methods used.

The new administration of WHO Director General Tedros Ghebreyesus has expressed a desire to make greater use of IHME outputs, particularly in relation to monitoring its Global Program of Work indicators and the SDG health indicators. An updated MOU between IHME and WHO was signed in 2018 [56], which envisages the two organizations moving to a single common GBD study, with an outline of how this is intended to be achieved. This will require the two organizations to address and resolve methodological and data differences between their differing approaches for substantial areas of the GBD. This will not be straightforward given that key health priorities are currently addressed by WHO through extensive participation in Interagency Groups, and that for some of these groups, another UN Organization acts as custodian in relation to the UN reporting on progress towards SDG targets.

Additionally, starting with the GBD 2017, the IHME is now producing its own population and birth estimates, which differ from those of the UN Population Division. This means that IHME estimates of numbers of deaths, both total and by cause, will differ systematically from those produced by UN agencies even when based on similar death rates. These differences are not always minor. When IHME first made its own birth estimates for the GBD2016 [57], it estimated there were 129 million births in 2016, 9 million less than the UN estimate of 138 million. More than half of this difference came from China alone where IHME estimated total births at 11.3 million compared to the UN estimate of 16.8 million. This resulted in a substantial drop in the IHME estimate of child deaths under 5 compared to the previous GBD2015, and a global total for child deaths in 2016 that was 600,000 fewer than the UN estimate of 5.6 million.

## Discussion

Since the end of the Second World War, WHO has had a unique mandate within international institutions as the official repository of international health data and analysis. However, WHO’s influence and ability to perform this role has been declining. Since the 1980s, the funding from Member States has been frozen in not only real but also nominal terms, and WHO has become increasingly reliant on voluntary contributions and grants, often for specific donor-driven objectives [58, 59]. Currently, around 80% of the overall WHO budget comes from such voluntary contributions and grants. The BMGF is now the second largest contributor to the WHO budget and the largest contributor, the US government, has been indicating a desire to reduce its contribution. The WHO is now in a situation where external donors can dictate priorities and policies in an increasing number of areas. At the same time, WHO has come under pressure to play an increased role in emergency and epidemic response, including direct on-the-ground involvement.

Additionally, the WHO position as the lead global health agency has been weakened by the emergence of a host of new actors including public private partnerships such as the Global Fund, GAVI and UNITAID and well-funded private bodies such as the BMGF, and the BMGF-funded IHME. The new administration of WHO appears keen to increase WHO reliance on IHME statistics and reduce WHO’s own activities in this area, which makes some sense in an era of declining funding and expanding mandates. IHME has substantial resourcing and expertise. However, it will also present a number of challenges.

Apart from the issue that IHME population estimates now differ from those used by the UN agencies, the complexity and computational intensity of IHME data imputation and modelling makes it extremely difficult for others to replicate or use their methods, or to explain how the outputs relate to country data. As Tichenor et al. [60] have noted in relation to an abortive attempt by the World Bank to use IHME’s work on a human capital index, the World Bank in the end decided to produce their own index so that they knew how it was derived from what data, and to have a platform to advocate for better data collection. An attempt by WHO on 2018 to switch from its own index of Universal Health Coverage to one developed by IHME [61] was rejected by the UN Inter-agency and Expert Group on SDG Indicators as it was deemed to be too removed from country data, with complex modelling and data imputation, to be acceptable to the UN Member States.

Many developing countries have little interest in the outputs of a project led by a US academic group and are very focused on WHO and UN statistics, particularly in relation to SDG targets. UN agencies have a mandate to produce statistics, some responsibility to consult with countries and are ultimately answerable to their Member States. Although the GBD project has thousands of collaborators, some involved in cause-specific data collection and analysis, others at country level providing inputs or advice, IHME performs most analysis centrally and retains control of analytic decision-making. IHME has argued that its independent academic status means it is less subject to political pressures than the UN agencies. The downside of IHME “independence” is that there have been quite drastic changes in methods and estimates from revision to revision for some causes and topics. Apart from the substantial change to Chinese child births and deaths noted above, another recent example is drug overdose deaths for USA, where GBD2016 excluded prescription opioid deaths without documenting this, but included them again in GBD2017. Global opioid dependence deaths went from an estimated 86,200 in GBD2016 to 109,500 in GBD2017, due to inclusion of prescription opioids, almost all the increase occurring in the USA.

The current administration does not seem concerned that WHO reports are publishing inconsistent statistics from IHME and from UN Interagency Groups. There is no longer a central coordination and clearance role within WHO for the production of coherent “UN” global health statistics. WHO Member States have already put pressure on WHO not to use IHME statistics for a universal health coverage index and I expect that difficulties in effectively collaborating with IHME will ultimately lead a future administration of WHO to seek to expand WHO expertise and work in the production of global health statistics once more.

A big unknown is the long-term impact of the coronavirus pandemic. On the one hand, IHME’s reputation has been tarnished by its development of a much criticised Covid-19 projection model [62, 63], whereas WHO has been able to work with leading infectious disease modelling groups across the world. On the other hand, a greatly increased focus of WHO on dealing with Covid 19 and future pandemics may reduce interest in addressing concerns about broader global health statistics.

## Conclusions

Over the last 20 years, WHO has undertaken substantial development and expansion of its work on global burden of disease and played a key role in harmonizing health statistics across relevant UN agencies through interagency collaborations. This has positioned WHO to play a lead role in the monitoring of global health trends for the Millenium Development Goals, and then the Sustainable Development Goals along with substantial support and advice to Member States on improvement of national health data.

For many Member States, the WHO still retains a unique mandate and accountability for global health statistics and a moral authority as a setter for norms and standards that is not available to academic or NGO groups. It remains to be seen whether WHO Member States will be comfortable with WHO lending its mandate for global health information to an independent North American academic group. It also remains to be seen whether WHO will continue to have the resources or will to carry out its own mandate in a world which at present seems to be increasingly turning away from multilateral global institutions, rules and norms.

## Data Availability

Not applicable.

## Abbreviations

BMGF: Bill and Melinda Gates Foundation
CHERG: WHO Child Health Epidemiology Reference Group
DALYs: Disability-Adjusted Life Years
DCPP: Disease Control Priorities Project
GATHER: Guidelines for Accurate and Transparent Health Estimates Reporting
GBD: Global Burden of Disease
GBD2010: Global Burden of Disease 2010 Study
GHE: WHO Global Health Estimates
IHME: Institute of Health Metrics and Evaluation
MDG: Millenium Development Goals
NBD: National Burden of Disease
NGO: Non-government Organization
RGHS: WHO Reference Group on Health Statistics
SDG: Sustainable Development Goals
UN: United Nations
UN-IGME: United Nations Interagency Group on Mortality Estimation
UNPD: United Nations Population Division
WHO: World Health Organization
YLD: Years Lost through Disability
YLL: Years of Life Lost
